# Diagnostic potential of TSH to HDL cholesterol ratio in vulnerable carotid plaque identification

**DOI:** 10.3389/fcvm.2024.1333908

**Published:** 2024-05-28

**Authors:** Meihua Lei, Shi-Ting Weng, Jun-Jun Wang, Song Qiao

**Affiliations:** ^1^ Department of Neurology, The Second Hospital of Jinhua, Jinhua, Zhejiang, China; ^2^ The Second Clinical Medical College, Zhejiang Chinese Medicine University, Hangzhou, Zhejiang, China; ^3^ Department of Neurology, Zhejiang Hospital, Hangzhou, Zhejiang, China

**Keywords:** carotid vulnerable plaque, component characteristics, thyroid-stimulating hormone, thyroid-stimulating hormone to high-density lipoprotein cholesterol ratio, high-resolution magnetic resonance imaging

## Abstract

**Objective:**

This study aimed to investigate the predictive value of the thyroid-stimulating hormone to high-density lipoprotein cholesterol ratio (THR) in identifying specific vulnerable carotid artery plaques.

**Methods:**

In this retrospective analysis, we included 76 patients with carotid plaques who met the criteria for admission to Zhejiang Hospital from July 2019 to June 2021. High-resolution magnetic resonance imaging (HRMRI) and the MRI-PlaqueView vascular plaque imaging diagnostic system were utilized to analyze carotid artery images for the identification of specific plaque components, including the lipid core (LC), fibrous cap (FC), and intraplaque hemorrhage (IPH), and recording of the area percentage of LC and IPH, as well as the thickness of FC. Patients were categorized into stable plaque and vulnerable plaque groups based on diagnostic criteria for vulnerable plaques derived from imaging. Plaques were categorized based on meeting one of the following consensus criteria for vulnerability: lipid core area over 40% of total plaque area, fibrous cap thickness less than 65 um, or the presence of intraplaque hemorrhage. Plaques meeting the above criteria were designated as the LC-associated vulnerable plaque group, the IPH-associated group, and the FC-associated group. Multivariate logistic regression was employed to analyze the factors influencing carotid vulnerable plaques and specific vulnerable plaque components. Receiver operating characteristic (ROC) curves were used to assess the predictive value of serological indices for vulnerable carotid plaques.

**Results:**

We found that THR (OR = 1.976; 95% CI = 1.094–3.570; *p* = 0.024) and TSH (OR = 1.939, 95% CI = 1.122–3.350, *p* = 0.018) contributed to the formation of vulnerable carotid plaques. THR exhibited an area under the curve (AUC) of 0.704 (95% CI = 0.588–0.803) (*p *= 0.003), and the AUC for TSH was 0.681 (95% CI = 0.564–0.783) (*p *= 0.008). THR was identified as an independent predictor of LC-associated vulnerable plaques (OR = 2.117, 95% CI = 1.064–4.212, *p* = 0.033), yielding an AUC of 0.815. THR also demonstrated diagnostic efficacy for LC-associated vulnerable plaques.

**Conclusion:**

This study substantiated that THR and TSH have predictive value for identifying vulnerable carotid plaques, with THR proving to be a more effective diagnostic indicator than TSH. THR also exhibited predictive value and specificity in the context of LC-associated vulnerable plaques. These findings suggest that THR may be a promising clinical indicator, outperforming TSH in detecting specific vulnerable carotid plaques.

## 1 Introduction

Stroke, characterized by its high incidence, disability, and recurrence, has become the leading cause of death in China, exerting a significant economic and medical burden on families and society (1). Despite guideline-based treatment, the risk of recurrent macrovascular events following ischemic stroke has remained consistently high at 25%–30% over the past five years (2). While stenosis serves as a valid susceptibility indicator for predicting the risk of ipsilateral cerebrovascular events, ongoing research has unveiled the limitations of relying solely on stenosis as the basis for determining the most effective preventive treatments. Interestingly, the composition characteristics of carotid atherosclerotic plaques are essential, especially for specific patient subpopulations with carotid atherosclerosis (3). Therefore, assessing the stability of carotid plaques is crucial. Accurate identification and characterization of vulnerable plaques and their components can significantly improve the prevention of adverse outcomes, such as cerebral infarction or transient ischemic attacks (TIA), and enable the selection of individualized treatment approaches for patients.

High-resolution magnetic resonance imaging (HRMRI) is currently the gold standard for examining carotid artery walls due to its exceptional soft-tissue resolution (4). The black blood sequence, a core technology of HRMRI, effectively suppresses blood flow signals within the carotid lumen and signals from surrounding adipose tissue, allowing for clear visualization of carotid plaque size, shape, and the differentiation of various components within the plaque, including the lipid core (LC), fibrous cap (FC), and intra-plaque hemorrhage (IPH), based on signal intensity differences (5). Bright blood techniques offer fast image acquisition with short echo/repetition times, providing the capability to assess FC and hemodynamic status (6). Studies have confirmed the high histopathological correlation of 3.0 T high-resolution magnetic resonance carotid plaque imaging (7). MRI-based plaque diagnostic systems, such as the MRI-PlaqueView vascular plaque imaging diagnostic system, have also been developed. After identifying plaque components, the system can reconstruct the 3D model and calculate it with its own measurement tools, and finally generate detailed reports, including area and volume percentages of different plaque components, degree of vascular stenosis, vessel thickness, and 3D plaque models, among other parameters. This vascular plaque imaging diagnostic system has demonstrated excellent sensitivity, specificity, and accuracy in identifying plaque composition (8).

While imaging methods can detect vulnerable plaques, they are associated with disadvantages such as time consumption, high costs, and radiation exposure. In contrast, serological indicators offer a cost-effective, rapid, and straightforward alternative. The past few years have witnessed a burgeoning interest in identifying serological markers for predicting vulnerable plaques. Recent research has indicated that the ratio of pro-inflammatory to anti-inflammatory factors could enhance the predictive value of vulnerable plaques. For instance, Sun M et al. and Liu HT et al. discovered that an elevated monocyte to high-density lipoprotein ratio (MHR) was independently associated with an increased risk of major adverse cardiovascular events (MACE) and higher all-cause mortality, making it a promising prognostic indicator for cardiovascular diseases (9, 10). Zhang Y et al. conducted a comparative study on MHR and monocyte count (MC) in predicting the prognosis of patients undergoing coronary angiography, revealing that MHR was independently linked to MACE and exhibited a prognostic effect similar to MC (11). Additionally, ratios such as C-reactive protein to high-density lipoprotein cholesterol, neutrophil to lymphocyte ratio, and platelet-to-lymphocyte ratio have shown similar effects in predicting cardiovascular events (12, 13). Building on this body of research, we endeavored to discover novel circulating markers to assist in diagnosing vulnerable carotid plaques and identifying specific vulnerable plaque components.

Recent studies have highlighted the association between thyroid-stimulating hormone and plaque vulnerability (14). TSH is known to increase the risk of atherosclerosis by regulating liver lipid metabolism, promoting endogenous cholesterol synthesis, thickening the vessel's inner media, inducing macrophage inflammation, and contributing to oxidative stress (15–17). Conversely, high-density lipoprotein cholesterol (HDL-C) plays a protective role in preventing atherosclerosis by promoting the reverse transport of cholesterol, inhibiting the oxidation of low-density lipoprotein cholesterol (LDL-C), and hindering monocyte migration and differentiation (18, 19). Given the contrasting effects of these two factors on atherosclerosis, we hypothesized that the thyroid-stimulating hormone to high-density lipoprotein cholesterol ratio (THR) may serve as a predictor of vulnerable carotid plaques. Furthermore, since vulnerable plaques comprise various components, the ability of THR to predict specific vulnerable plaque components remains unexplored. Herein, we sought to determine whether THR can be employed as a simple, cost-effective, and convenient indicator for predicting carotid plaque vulnerability and its specific components, necessitating further clinical investigations to substantiate this hypothesis.

## 2 Methods

### 2.1 Eligibility criteria

This study constitutes a single-center cross-sectional investigation. It encompassed patients admitted to Zhejiang Hospital for their initial carotid HRMRI examination from July 2019 to June 2021. Approval for this study was obtained from the Zhejiang Hospital Ethics Committee. Inclusion criteria comprised: (1) age equal to or greater than 18 years; (2) evidence of carotid atherosclerotic plaque on carotid ultrasound; (3) confirmation of carotid atherosclerotic plaques through further MRI examination; and (4) complete medical records for the patient. The main exclusion criteria included: (1) patients with severe cardiovascular conditions (acute coronary syndrome), hematological disorders, and liver or kidney dysfunction; (2) individuals with severe infection, sepsis, malignancies, autoimmune diseases, or those taking immunosuppressants, glucocorticoids, or cytotoxic medications; (3) patients admitted following carotid endarterectomy or carotid artery stenting; (4) individuals who had undergone intravenous thrombolysis or interventional therapy; and (5) cases where HRMRI image quality was suboptimal.

### 2.2 Clinical data collection

Baseline data were collected from study participants, covering age, gender, body mass index (BMI), history of smoking, history of alcohol consumption, presence of hypertension and/or diabetes, prior history of stroke, atrial fibrillation, history of statin utilization, and a history of hyperthyroidism or hypothyroidism. Additionally, clinical laboratory indicators were collected at baseline, including white blood cell count (WBC), neutrophils, lymphocytes, C-reactive protein (CRP), D-dimer, uric acid, homocysteine (Hcy), alanine aminotransferase, aspartate aminotransferase, low-density lipoprotein cholesterol (LDL-C), HDL-C, triglycerides (TG), total cholesterol (TC), triiodothyronine, total thyroxine, and TSH. The thyroid-stimulating hormone to high-density lipoprotein cholesterol ratio was calculated.

### 2.3 Image acquisition

Images were acquired using 3 T MR imaging scanners (Siemens Syera, Germany) equipped with standard head/neck coils. During the examination, the patient's head and neck were securely positioned, and patients were instructed to remain still and minimize swallowing movements. Scanning of the carotid bifurcation was conducted 3 cm above and below, centered on 3D-TOF self-reconstituted MRA. Notably, no contrast agents were employed, ensuring a completely noninvasive technique for carotid plaque visualization. For signal intensity comparisons of carotid plaques on T1-weighted imaging, T2-weighted imaging, and time-of-flight sequences, signals from adjacent muscle tissues were considered equal and utilized as a reference. The HRMRI sequence parameters are detailed in Table 1.

**Table 1:** Sequence parameters of high resolution magnetic resonance.

Sequence parameter	T1WI	T2WI	TOF
Impulse sequence	TSE	TSE	FFE
TR/TE, ms	800/10	4,800/50	20/4
FOV, cm	16 × 16	16 × 16	16 × 16
Matrix	256 × 256	256 × 256	256 × 256
FA	90°	90°	20°
Thick, mm	2	2	1

T1WI, T1-weighted imaging; T2WI, T2-weighted imaging; TOF, time of flight; TR, repetition time; TE, echo time; FOV, field of vision; FA, flip angle.

### 2.4 Imaging analysis

**Figure 1:**
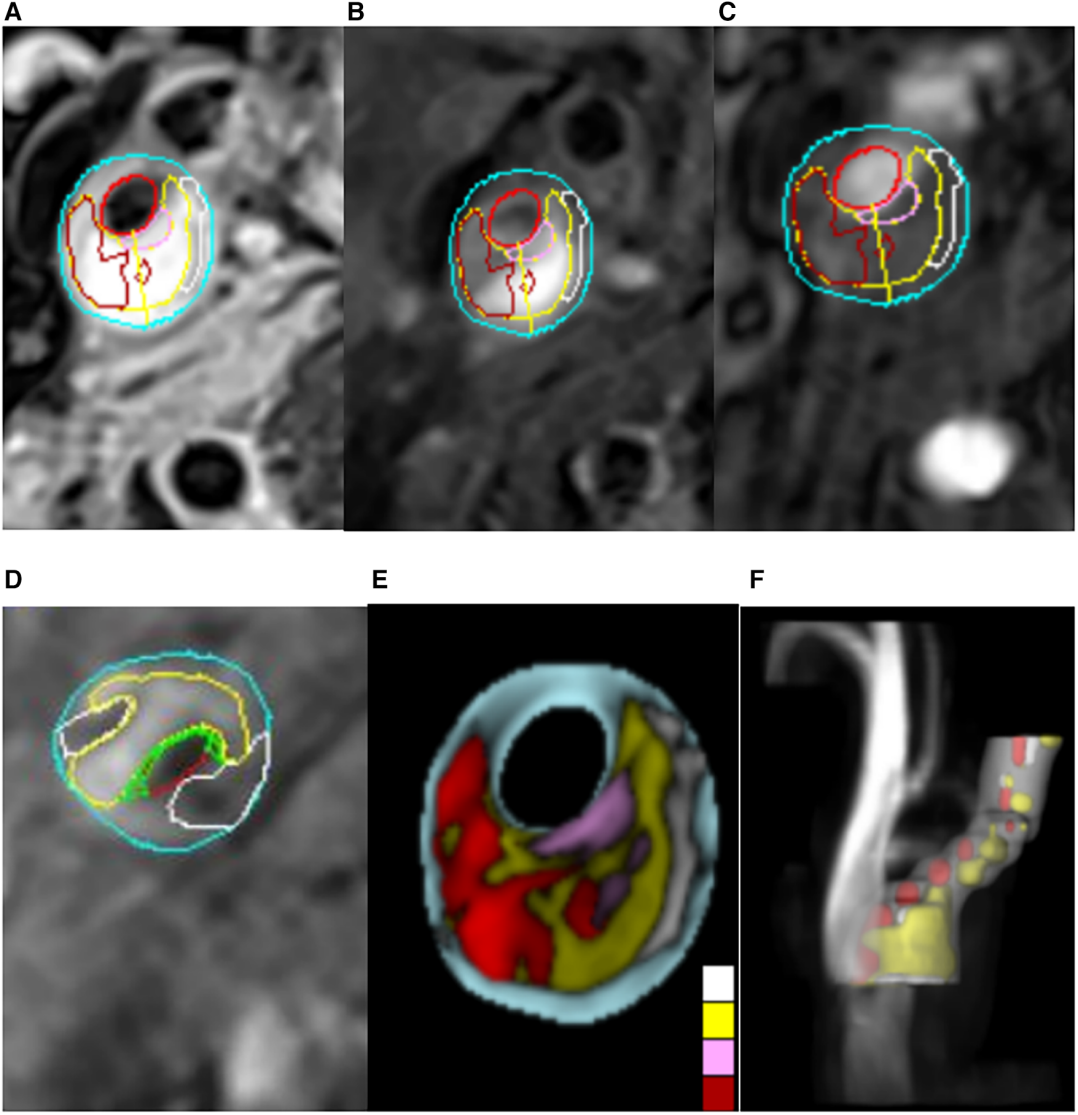
Carotid MRI semi-automatic segmentation of plaque composition. (**A–D**) The MRI-PlqueView vascular plaque imaging Diagnostic System was used to delineate the plaque component regions on T1-weighted imaging (T1WI), T2-weighted imaging (T2WI) and time-of-flight (TOF) sequences. Different colors indicated different plaque components. Yellow represents lipids, white represents calcification, green represents fibrous caps, dark red represents intraplastic hemorrhage, positive red represents lumen, and blue represents wall. (**E,F**) The MRI-PlaqueView vascular plaque imaging Diagnostic System was used to automatically recompose the cross section and 3D blood vessel images.

All image groups were processed using plaque analysis software (MRI-Plaque View, VPDiagnostics). Two radiologists, with 3 and 9 years of experience respectively in magnetic resonance characterization of carotid plaque, dissected the plaques and performed blind image reviews of plaque components. To address inconsistencies in the number of layers, thickness, and motion between scans from the two imaging sequences, position matching was conducted for each artery image including 3D TOF image reconstruction. Quantitative measurements of lumen and wall areas of bilateral carotid arteries were obtained on T2WI. Identification standards for IPH, LRNC, and FC were determined based on signal standards from conventional scanning protocols. The patch component area at each layer was manually delineated while the software's built-in measurement tool quantified the percentage area occupied by patch components and FC thickness at each layer (Figure 1). As per the diagnostic criteria for vulnerable plaques (20, 21), the following conditions indicated vulnerable plaques: FC thickness <65 um or LC accounting for more than 40% of the plaque area or the presence of IPH. The diagnostic system demonstrated high sensitivity, specificity, and accuracy in identifying plaque components (8, 22). Plaques were categorized according to the American Heart Association (AHA) plaque typing criteria (23), wherein type IV, V, and VI plaques were classified as vulnerable, while type I–II, III, VII, and VIII plaques were categorized as stable. The consistency test revealed strong internal consistency within the groups (Kappa = 0.919, *p* < 0.001).

### 2.5 Statistical analysis

Statistical analysis was carried out using SPSS 25.0 and MedCalc 19.0.4 software. The Kolmogorov-Smirnov test was employed to assess the normality of the distribution of continuous variables. For normally distributed continuous variables, baseline data were expressed as mean ± standard deviation (SD). Categorical variables were presented as percentages (%). The two independent samples *t*-test and Mann-Whitney *U*-test were utilized for comparing normally and non-normally distributed measured data, respectively. The Chi-square test was employed for comparing categorical variables. To determine the independent predictors of vulnerable carotid plaques, multivariate logistic regression analysis was performed for parameters with significant values in the univariate analysis (*p* < 0.05). Odds Ratio (OR) and 95% Confidence Interval (CI) were calculated. Spearman linear correlation analysis assessed the correlation between variables and specific vulnerable plaque components. Receiver Operating Characteristic (ROC) curve analysis was used to calculate the area under the curve (AUC) for vulnerable plaques, and diagnostic efficacy was assessed using sensitivity, specificity, positive predictive value, negative predictive value, and diagnostic accuracy. A two-tailed *p*-value of <0.05 was statistically significant.

## 3 Results

### 3.1 General data analysis

In the present study, a total of 90 patients were initially admitted for carotid plaque evaluation. However, patients were excluded due to severe infection (*n* = 5), prior carotid artery stenting (*n* = 4), and suboptimal image quality (*n* = 5). Thus, the final cohort comprised 76 patients with a mean age of (71.57 ± 10.32) years. Of these, 30 patients were classified into the stable plaque group and 46 into the vulnerable plaque group. Within the stable plaque group, 17 individuals were male (56.70%), with a mean age of (70.17 ± 11.58) years, while the vulnerable plaque group comprised 37 males with a mean age of (72.48 ± 9.43) years. A significantly higher prevalence of vulnerable plaques was observed in males (*p* = 0.026). However, there were no significant differences between the two groups in terms of age, BMI, smoking history, alcohol consumption history, history of hypertension, diabetes, stroke, atrial fibrillation, hypothyroidism and statin utilization (*p *> 0.05) (Table 2).

**Table 2:** Comparison of general data between vulnerable plaque and stable plaque.

Items	Stable plaque (*n* = 30)	Vulnerable plaque (*n* = 46)	χ^2^/*t* value	*p*-value
Age, years	70.17 ± 11.58	72.48 ± 9.43	−0.954	0.343
Male, *n* (%)	17 (56.7)	37 (80.4)	4.987	0.026
Smoking, *n* (%)	7 (23.3)	16 (34.8)	1.128	0.288
Drinking, *n* (%)	5 (16.7)	14 (30.4)	1.836	0.175
Hypertension, *n* (%)	19 (63.3)	36 (78.3)	2.023	0.155
Diabetes, *n* (%)	7 (23.3)	11 (23.9)	0.003	0.954
BMI, kg/m^2^	23.13 ± 3.62	24.45 ± 3.17	−1.648	0.104
History of stroke, *n* (%)	14 (46.7)	18 (39.1)	0.423	0.515
AF, *n* (%)	2 (6.7)	6 (13.0)	0.253	0.615
Hypothyroidism, *n* (%)	1 (3.3)	9 (19.6)	2.887	0.089
History of statin utilization, *n* (%)	9 (30.0)	13 (28.3)	0.027	0.870

BMI, body mass index; AF, atrial fibrillation.

### 3.2 Laboratory data analysis

Statistical analysis of laboratory data showed significant differences in D-dimer, Hcy, LDL-C, TG, TC, and TSH between the two groups (*p* < 0.05) (Table 3). After adjusting for confounding factors using multivariate logistic regression analysis, it was found that elevated levels of TSH (OR = 1.939, 95% CI = 1.122–3.350, *p* = 0.018) and THR (OR = 1.976, 95% CI = 1.094–3.570, *p* = 0.024) were influential factors for vulnerable carotid plaques (Tables 4, 5).

**Table 3:** Comparison of laboratory indices between vulnerable plaque and stable plaque.

Items	Stable plaque (*n* = 30)	Vulnerable plaque (*n* = 46)	*t*/*z* value	*p*-value
WBC (×10^9^/L)	6.63 ± 2.15	6.25 ± 1.84	0.820	0.415
Neutrophils (×10^9^/L)	4.47 ± 1.87	4.12 ± 1.55	0.892	0.375
Lymphocyte (×10^9^/L)	1.66 ± 0.75	1.49 ± 0.50	1.174	0.244
UA, umol/L	336.77 ± 100.98	345.89 ± 87.49	−0.418	0.677
TG,mmol/L	1.38 ± 0.57	1.10 ± 0.41	2.525	0.014
TT3, ng/ml	0.98 ± 0.23	0.99 ± 0.22	−0.186	0.853
TT4, ug/dl	8.27 ± 1.88	8.43 ± 1.86	−0.361	0.719
CRP (×10^9^/L)	1.44 (0.77, 3.07)	2.22 (0.80, 4.98)	−1.374	0.169
D-dimer, mg/L	0.25 (0.22, 0.37)	0.35 (0.22, 0.55)	−1.970	0.049
Hcy, umol/L	10.76 (9.16, 14.93)	13.55 (11.19, 16.35)	−2.274	0.023
ALT, U/L	20.50 (13.75, 28.25)	19.00 (14.50, 28.00)	−0.011	0.992
AST, U/L	22.50 (15.75, 31.50)	23.00 (17.00, 28.00)	−0.378	0.706
LDL-C, mmol/L	2.33 (1.82, 2.78)	1.81 (1.56, 2.27)	−2.264	0.024
HDL-C, mmol/L	1.14 (1.01, 1.35)	1.05 (0.92, 1.27)	−1.499	0.134
TC, mmol/L	3.94 (3.47, 4.67)	3.37 (2.92, 4.02)	−2.184	0.029
TSH, uIU/ml	1.54 (0.80, 2.21)	2.08 (1.31, 3.82)	−2.646	0.008
THR (%)	1.40 ± 0.90	2.44 ± 1.70	−3.079	0.003

The skewed distribution was tested by rank sum test and expressed as 50% (25%, 75%).

WBC, white blood cell; CRP, C-reactive protein; UA, uric acid; Hcy, homocysteine; AST, alanine aminotransferase; ALT, aspartate aminotransferase; LDL-C, low-density lipoprotein cholesterol; HDL-C, high-density lipoprotein cholesterol; TG, triglyceride; TC, total cholesterol; TT3, triiodothyronine; TT4, total thyroxine; TSH, thyrotropin; THR, thyroid-stimulating hormone and high-density lipoprotein cholesterol ratio.

**Table 4:** Multivariate logistic regression analysis of the influence of clinical indicators on vulnerable plaques.

Variables	OR	95% CI	*p*-value
TSH	1.939	1.122–3.350	0.018
TG	0.350	0.097–1.264	0.109
D-dimer	11.877	0.389–362.732	0.156
Male	2.201	0.628–7.709	0.218
Hcy	1.095	0.983–1.219	0.099
LDL-C	0.494	0.139–1.753	0.275
TC	1.045	0.363–3.010	0.934

TSH, thyrotropin; TG, triglyceride; Hcy, homocysteine; LDL-C, low-density lipoprotein cholesterol; TC, total cholesterol.

**Table 5:** Multivariate logistic regression analysis of the influence of clinical indicators on vulnerable plaques.

Variables	OR	95% CI	*p*-value
THR	1.976	1.094–3.570	0.024
TG	0.317	0.089–1.131	0.077
D-dimer	11.816	0.402–347.394	0.152
Male	2.047	0.597–7.018	0.254
Hcy	1.091	0.980–1.214	0.112
LDL-C	0.460	0.131–1.621	0.227
TC	1.155	0.408–3.268	0.786

THR, thyroid-stimulating hormone and high-density lipoprotein cholesterol ratio; TG, triglyceride; Hcy, homocysteine; LDL-C, low-density lipoprotein cholesterol; TC, total cholesterol.

### 3.3 Diagnostic efficacy of THR and TSH in vulnerable plaques

**Figure 2:**
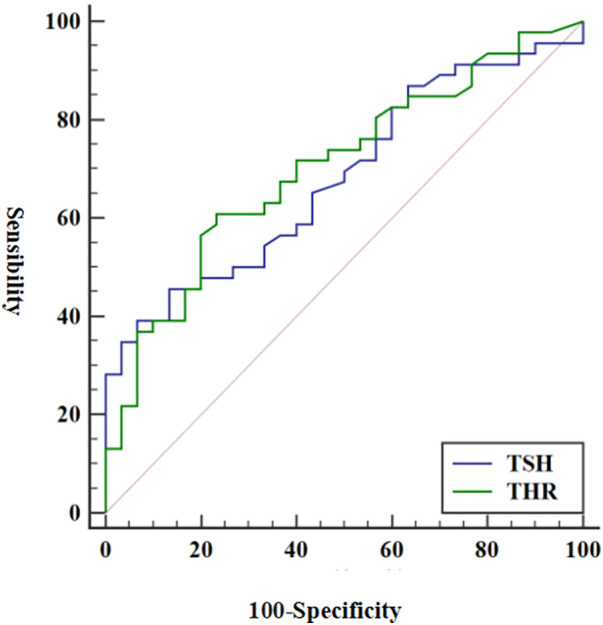
ROC of THR and TSH in diagnosing vulnerable plaques. Using vulnerable plaques as reference criteria, the AUC values of THR and TSH for predicting vulnerable plaques were 0.704 and 0.681, respectively (*p *< 0.05). ROC, receiver operating characteristic; THR, thyroid-stimulating hormone and high-density lipoprotein cholesterol ratio; TSH, thyrotropin; AUC, area under the curve.

The predictive abilities of THR and TSH for vulnerable plaques are illustrated in Figure 2. The AUC value for THR was 0.704 (95% CI = 0.588–0.803) (*p *= 0.003), while the AUC value for TSH was 0.681 (95% CI = 0.564–0.783) (*p *= 0.008). The diagnostic parameters describing the predictive performance of THR and TSH are presented in Table 6. THR exhibited slightly higher diagnostic efficiency than TSH, with superior sensitivity.

**Table 6:** Diagnostic efficacy of THR and TSH in vulnerable plaques.

	THR	TSH
True positive	28	18
True negative	23	28
False positive	7	2
False negative	18	28
Sensibility	60.87	39.13
Specificity	76.67	93.33
Positive predictive value	80.00	90.00
Negative predictive value	56.09	50.00
Diagnostic accuracy	67.11	60.53
AUC (95% CI)	0.704 (0.588–0.803)	0.681 (0.564–0.783)

THR, thyroid-stimulating hormone and high-density lipoprotein cholesterol ratio; TSH, thyrotropin; AUC, area under the curve.

### 3.4 Analysis of influencing factors of serological indexes on specific vulnerable plaque components

To investigate serological indicators influencing the formation of vulnerable plaques by specific components, 14 vulnerable plaques with LC area >40% were categorized into the LC-associated vulnerable plaque group (*n* = 46), while 12 vulnerable plaques with only IPH were grouped into the IPH-associated vulnerable plaque category. Additionally, seven vulnerable plaques with FC thickness <65 um were classified as FC-associated vulnerable plaques. Specific serological indicators were chosen and compared with the stable plaque group. The results of single-factor analysis are displayed in Tables 7–9. Serological indicators with *P* < 0.1 in the aforementioned univariate analysis were included in multivariate logistic regression (Table 10), which revealed that THR was an independent influencing factor for LC-associated vulnerable plaques (OR = 2.117, 95% CI = 1.064–4.212, *p* = 0.033). D-dimer was identified as an independent factor affecting IP-related vulnerable plaques (OR = 2,028,875.621, 95% CI = 4.972–2.272e + 11, *p* = 0.028), while there were no significant differences for the other indicators (*p* > 0.05).

**Table 7:** Comparison of serological indices between stable plaque group and LC-associated vulnerable plaque group.

	Stable plaque group (*n* = 30)	LC-associated vulnerable plaque group (*n* = 14)	*t*/*z* value	*p*-value
TG, mmol/L	1.38 ± 0.57	1.33 ± 0.39	0.345	0.732
CRP (×10^9^/L)	1.44 (0.77, 3.07)	3.21 (0.72, 4.95)	−0.983	0.326
D-dimer, mg/L	0.25 (0.22, 0.37)	0.26 (0.21, 0.48)	−0.354	0.732
LDL-C, mmol/L	2.33 (1.82, 2.78)	2.32 (2.09, 3.22)	−0.718	0.473
HDL-C, mmol/L	1.14 (1.01, 1.35)	0.97 (0.87, 1.02)	−3.088	0.002
TC, mmol/L	3.94 (3.47, 4.67)	3.98 (3.10, 4.94)	−0.491	0.623
TSH, uIU/ml	1.54 (0.80, 2.21)	2.30 (1.45, 3.42)	−1.613	0.107
THR (%)	1.40 ± 0.90	2.98 ± 1.74	−3.983	<0.001

LC, lipid core; CRP, C-reactive protein; LDL-C, low-density lipoprotein cholesterol; HDL-C, high-density lipoprotein cholesterol; TC, total cholesterol; TSH, thyrotropin; THR, thyroid-stimulating hormone and high-density lipoprotein cholesterol ratio. The skewed distribution was tested by rank sum test and expressed as 50% (25%, 75%).

**Table 8:** Comparison of serological indices between stable plaque group and IPH-associated vulnerable plaque group.

	Stable plaque group (*n* = 30)	IPH-associated vulnerable plaque group (*n* = 12)	*t/z* value	*p*-value
TG, mmol/L	1.38 ± 0.57	0.86 ± 0.32	2.968	0.005
CRP (×10^9^/L)	1.44 (0.77, 3.07)	2.13 (0.86, 9.01)	−0.983	0.326
D-dimer, mg/L	0.25 (0.22, 0.37)	0.52 (0.28, 1.43)	−2.580	0.010
LDL-C, mmol/L	2.33 (1.82, 2.78)	1.71 (1.20,1.83)	−2.646	0.008
HDL-C, mmol/L	1.14 (1.01, 1.35)	1.20 (1.03, 1.40)	−0.139	0.889
TC, mmol/L	3.94 (3.47, 4.67)	3.07 (2.74, 3.81)	−2.423	0.015
TSH, uIU/ml	1.54 (0.80, 2.21)	2.06 (0.86, 4.57)	−1.295	0.195
THR (%)	1.40 ± 0.90	2.52 ± 2.20	−1.702	0.114

IPH, intraplastic hemorrhage; CRP, C-reactive protein; LDL-C, low-density lipoprotein cholesterol; HDL-C, high-density lipoprotein cholesterol; TC, total cholesterol; TSH, thyrotropin; THR, thyroid-stimulating hormone and high-density lipoprotein cholesterol ratio. The skewed distribution was tested by rank sum test and expressed as 50% (25%, 75%).

**Table 9:** Comparison of serological indices between stable plaque group and FC-associated vulnerable plaque group.

	Stable plaque group (*n* = 30)	FC-associated vulnerable plaque group (*n* = 7)	*t/z* value	*p*-value
TG, mmol/L	1.38 ± 0.57	1.27 ± 0.33	0.484	0.632
CRP (×10^9^/L)	1.44 (0.77, 3.07)	1.93 (0.53, 3.24)	−0.136	0.892
D-dimer, mg/L	0.25 (0.22, 0.37)	0.36 (0.31, 0.47)	−1.691	0.091
LDL-C, mmol/L	2.33 (1.82, 2.78)	1.78 (1.48, 1.93)	−1.959	0.050
HDL-C, mmol/L	1.14 (1.01, 1.35)	1.02 (0.90, 1.16)	−1.435	0.151
TC, mmol/L	3.94 (3.47, 4.67)	3.07 (2.61, 3.43)	−2.676	0.007
TSH, uIU/ml	1.54 (0.80, 2.21)	1.29 (0.60, 3.80)	−0.310	0.756
THR (%)	1.40 ± 0.90	1.71 ± 1.21	−0.760	0.453

FC, fiber cap; CRP, C-reactive protein; LDL-C, low-density lipoprotein cholesterol; HDL-C, high-density lipoprotein cholesterol; TC, total cholesterol; TSH, thyrotropin; THR, thyroid-stimulating hormone and high-density lipoprotein cholesterol ratio. The skewed distribution was tested by rank sum test and expressed as 50% (25%, 75%).

**Table 10:** Multivariate logistic regression of stable plaque group and specific vulnerable plaque group.

	Serological indicators	OR	95% CI	*p*-value
LC-associated vulnerable plaque group	THR	2.117	1.064–4.212	0.033
	HDL-C	0.017	<0.001–2.541	0.110
IPH-associated vulnerable plaque group	LDL-C	0.043	0.001–1.754	0.096
	TG	0.022	<0.001–1.351	0.069
	TC	2.719	0.181–40.956	0.470
	D-dimer	2,027,875.621	4.972–2.272E + 11	0.028
FC-associated vulnerable plaque group	LDL-C	1.995	0.078–51.289	0.677
	TC	0.107	0.007–1.541	0.100
	D-dimer	514.029	0.098–2,683,429.879	0.153

LC, lipid core; IPH, intraplastic hemorrhage; FC, fiber cap; LDL-C, low-density lipoprotein cholesterol; HDL-C, high-density lipoprotein cholesterol; TC, total cholesterol; TG, triglyceride; THR, thyroid-stimulating hormone and high-density lipoprotein cholesterol ratio.

### 3.5 Diagnostic efficacy of THR for LC-associated vulnerable plaques

**Figure 3:**
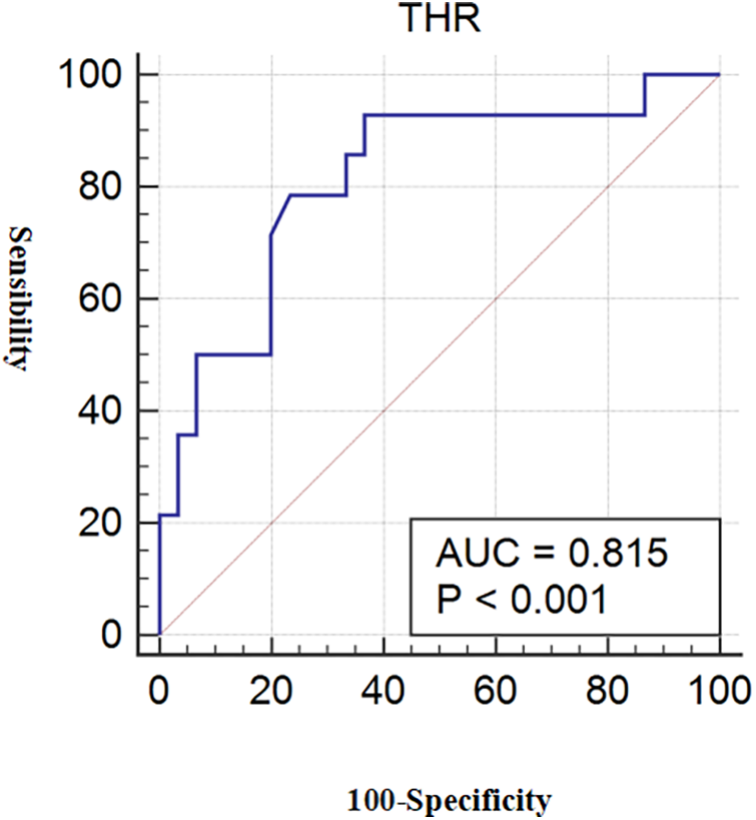
ROC for THR diagnosis of LC-associated vulnerable plaques. With LC area >40% as the reference standard for vulnerable plaques, the AUC value of THR for predicting LC-associated vulnerable plaques were 0.815 (*p* < 0.001). ROC, receiver operating characteristic; THR, thyroid-stimulating hormone and high-density lipoprotein cholesterol ratio; LC, lipid core; AUC, area under the curve.

The predictive capacity of THR for LC-associated vulnerable plaques is depicted in Figure 3. THR displayed an AUC of 0.815 (95% CI = 0.670–0.916) (*p* < 0.001). Diagnostic parameters characterizing the predictive performance of THR are outlined in Table 11, demonstrating THR's predictive value and specificity for LC-associated vulnerable plaques.

**Table 11:** Diagnostic efficacy of THR for LC-associated vulnerable plaques.

	THR
True positive	13
True negative	19
False positive	11
False negative	1
Sensibility	92.86
Specificity	63.33
Positive predictive value	54.17
Negative predictive value	95.00
Diagnostic accuracy	72.73
AUC (95% CI)	0.815 (0.670–0.916)

THR, thyroid-stimulating hormone and high-density lipoprotein cholesterol ratio; LC, lipid core; AUC, Area under the curve.

## 4 Discussion

Vulnerable carotid artery plaques pose a significant risk of acute cardiovascular and cerebrovascular events, leading to poor outcomes and even death. Therefore, developing tools for diagnosing and identifying vulnerable plaques is essential. While imaging methods such as ultrasound and MRI are commonly used in clinical practice to assess carotid plaques, they have limitations, including time-consuming procedures, high costs, patient tolerance issues, and the need for experienced medical personnel. This restricts the early detection and intervention of vulnerable carotid plaques. Accordingly, there has been a growing interest in using serological indicators to predict vulnerable carotid plaques due to their wide coverage, rapid availability, cost-effectiveness, and simplicity.

### 4.1 The pathological mechanism of THR in predicting vulnerable carotid plaque

Hypothyroidism has been well recognized to be accompanied by hypercholesterolemia and cardiovascular diseases. Traditionally, this is attributed to the decreased thyroid hormone levels in these patients. However, increased risk of hypercholesterolemia and cardiovascular diseases including AS are also found among subclinical hypothyroidism patients, whose thyroid hormone levels remain normal and only TSH levels are increased (24). This suggests that TSH may also play roles in atherosclerosis independent from its influence on thyroid hormones. Therefore, we gradually focused on TSH. THR, a new indicator, represents the ratio of TSH to HDL-C. THR's effect and predictive value in assessing vulnerable carotid plaques may be attributed to the involvement of TSH and HDL-C in the body's inflammatory and metabolic processes. The results of the present study indicated that, in addition to THR, TSH is also a significant factor in the formation of carotid artery vulnerable plaques and holds predictive value. Furthermore, THR outperformed TSH in terms of diagnostic efficacy and sensitivity. Several factors may explain the mechanism through which elevated TSH contributes to plaque instability: (1) Direct Regulation of Liver Lipid Metabolism: TSH plays a pivotal role in regulating liver lipid metabolism (14, 25). Typically, TSH binds to its receptor, the Thyroid Stimulating Hormone Receptor (TSHR), not only in the thyroid but also in various extra-thyroidal tissues, including liver cells, osteoclasts, adipocytes, and macrophages (16). Research indicates that TSHR is functionally expressed on the surface of liver cells. When TSH binds to its receptor, it stimulates the release of cyclic adenosine phosphate (cAMP), which, in turn, upregulates the expression of HMG-CoA reductase, a key enzyme in cholesterol synthesis, through the cAMP/PKA/CREB pathway. This process leads to increased synthesis of endogenous cholesterol (26). Concurrently, elevated TSH levels reduce lipoprotein lipase activity, decreasing the efficiency of cholesterol conversion into bile acids and significantly inhibiting lipid clearance. Consequently, total cholesterol (TC) and triglyceride (TG) levels increase (16). Elevated TG-rich lipoproteins are widely acknowledged to promote inflammatory activities and contribute to atherosclerosis (AS) (27). Human studies have shown a positive correlation between elevated serum TSH levels and increased atherosclerotic lipid profiles, even within the normal reference range. Additionally, thyroid hormone replacement therapy, which reduces TSH levels, has been associated with lower blood lipid levels and a delayed progression of AS (28). Another study showed that serum TSH levels were positively correlated with cholesterol, even within the normal reference range, which also validated the influence of TSH on lipid metabolism (16). (2) Promotion of Atherosclerosis by Thickening Endovascular Media: TSHR is expressed in key cell types relevant to atherosclerosis, including macrophages, endothelial cells, and vascular smooth muscle cells (VSMCs). TSH activates the expression of factors associated with vascular endothelial function and promotes the proliferation of VSMCs, leading to thickening of blood vessel walls. Furthermore, VSMCs release extracellular matrix components and cytokines, increasing lipid content within plaques (29–31). Studies have demonstrated that populations with elevated TSH levels significantly increase Intima-Media Thickness (IMT) in arterial blood vessels, including carotid IMT and aortic IMT. Lipid infiltration into arterial walls is considered the primary mechanism behind this elevated IMT (32, 33). (3) Direct Stimulation of Macrophage Inflammatory Response: TSH can reportedly activate the MAPK (ERK1/2, P38, JNK) and I kappa B/P65 pathways in macrophages. This activation results in enhanced production of inflammatory cytokines, including tumor necrosis factor (TNF)-α and Interleukin (IL)-6, thus promoting an inflammatory response within the body and encouraging monocyte recruitment (34–36). TSH also induces the generation of Reactive Oxygen Species (ROS), contributing to oxidative stress and apoptosis and ultimately facilitating the development of vulnerable plaques (16, 17). In experiments conducted by Professor Zhao Jiajun's team to determine that the occurrence of AS and the above inflammatory reactions are indeed caused by TSH, two groups of hybrid mice were fed, one with intact TSHR and the other with TSHR knockout, under identical conditions. After a period of observation, results from *in vitro* staining indicated that mice with TSHR knockout exhibited a significant reduction in plaque area and a decreased number of macrophages. Importantly, vascular inflammation was attenuated, with decreased expression of inflammatory factors, including TNF-α and IL-6 (16). These findings provide strong evidence that TSH plays a role in promoting vascular inflammation and atherosclerosis.

The predictive value of THR for vulnerable carotid plaque was marginally superior to that of TSH, which may be attributed to the role of HDL-C. It is now understood that HDL-C possesses anti-inflammatory properties and can prevent the development of vulnerable plaque, rendering it a protective factor for carotid plaque. Its anti-inflammatory mechanisms include: (1) inhibition of Low-Density Lipoprotein Cholesterol (LDL-C) oxidation; (2) suppression of endothelial cell adhesion molecule production induced by cytokines; (3) effective reduction of monocyte chemotactic protein-1 secretion from oxidized low-density lipoprotein in endothelial cells; and (4) facilitation of cholesterol outflow from mononuclear macrophage-derived foam cells (18, 19, 37). Although our study did not establish the influence of HDL-C on vulnerable plaque, it did reveal that THR has become an influential factor in vulnerable carotid artery plaque. Based on our findings, it is highly conceivable that THR might reflect the imbalance between TSH and HDL-C in plaque formation, thereby serving as a superior predictor of vulnerable plaque compared to TSH and HDL-C.

### 4.2 THR is specific for the diagnosis of vulnerable carotid plaque associated with LC

Specific plaque components constitute essential criteria for diagnosing plaque vulnerability, including LC area >40%, fiber cap thickness <65 um, and the presence of IPH (20, 21). Limited research has explored the prediction of specific plaque composition. Herein, we aimed to explore the relationship between LC, IPH, FC-related vulnerable plaques, and THR and determine diagnostic efficiency. The results indicated that THR significantly influenced LC-associated vulnerable plaques, whereas no notable distinction was observed for the other two components. Moreover, using LC-associated vulnerable plaques as the reference standard, ROC curve analysis demonstrated the predictive value of THR in LC-associated carotid artery vulnerable plaques (AUC value 0.815, sensitivity 92.86%, specificity 63.33%). These findings suggest that THR may diagnose LC-associated vulnerable carotid plaque. As mentioned above, it is highly conceivable that this specificity is linked to the involvement of TSH and HDL-C in the lipid metabolism of vulnerable plaques. However, TSH and HDL-C levels exhibited limited ability to predict lipid core-associated vulnerable plaques, implying that THR may be more sensitive than TSH and HDL-C. To validate our results and explore the potential of THR in predicting more specific vulnerable carotid plaques, it is imperative to expand the sample size and conduct prospective studies in the future.

This study has several limitations that should be acknowledged. Firstly, it is a retrospective single-center study, necessitating further large-scale prospective multicenter studies to validate our findings. Secondly, although this study employed HRMRI in conjunction with a plaque diagnosis system to differentiate plaque components and enhance detection accuracy, potential artifacts and errors resulting from software characterizing plaque components could not be eliminated. Thirdly, the lack of follow-up data precluded the evaluation of the significance of THR and TSH in guiding the diagnosis, treatment, and prognosis of patients with carotid plaque. Future studies should further investigate the efficacy of THR, with a focus on vascular events as the primary endpoint. Finally, THR has yet to be implemented in clinical practice, and intervention measures have not been employed to verify its efficacy in LC-associated vulnerable plaques, nor has the effect of THR on other vulnerable plaque components been fully defined. Given that THR is a novel indicator for predicting specific vulnerable plaques, extensive clinical studies are warranted to confirm its utility in subsequent stages.

## 5 Conclusion

Overall, THR and TSH exhibit good predictive value for vulnerable carotid plaques. THR's diagnostic performance was superior to TSH, suggesting that it could serve as a novel circulating marker for identifying vulnerable plaques, especially for LC-associated vulnerable plaques. As a novel and promising indicator, THR enables early identification and targeted intervention for patients with LC-associated vulnerable plaque, facilitating diagnostic and treatment decision-making for clinicians.

## Data Availability

The raw data supporting the conclusions of this article will be made available by the authors, without undue reservation.
